# A global bibliometric and visualized analysis of the links between the autophagy and acute myeloid leukemia

**DOI:** 10.3389/fphar.2023.1291195

**Published:** 2024-01-23

**Authors:** Yao Gao, Zhenhui Wu, Yingfan Chen, Guangbin Shang, Yingjian Zeng, Yue Gao

**Affiliations:** ^1^ Graduate School, Jiangxi University of Chinese Medicine, Nanchang, China; ^2^ Affiliated Hospital of Jiangxi University of Chinese Medicine, Nanchang, China; ^3^ Department of Traditional Chinese Medicine, Sixth Medical Center, PLA General Hospital, Beijing, China; ^4^ Beijing Institute of Radiation Medicine, Beijing, China

**Keywords:** acute myeloid leukemia, autophagy, bibliometric analysis, chemotherapy resistance, mitophagy

## Abstract

**Background and objectives:** Autophagy is a cellular process where damaged organelles or unwanted proteins are packaged into a double-membrane structure and transported to lysosomes for degradation. Autophagy plays a regulatory role in various hematologic malignancies, including acute myeloid leukemia (AML). However, there are few bibliometric studies on the role of autophagy in AML. The purpose of this study is to clarify the role of autophagy in acute myeloid leukemia through bibliometric analysis.

**Methods:** The literature on autophagy and AML research from 2003 to 2023 was searched in Web of Science Core Collection, and bibliometric tools such as VOSviewer 1.6.18, Cite Space (6.1.R3), RStudio (R package bibliometrix), and Scimago Graphica were used to understand the current status and hotspots of autophagy and AML research. The study conducted an analysis of various dimensions including the quantity of publications, countries, institutions, journals, authors, co-references, keywords, and to predict future development trends in this field by drawing relevant visualization maps.

**Results:** A total of 343 articles were obtained, published in 169 journals, written by 2,323 authors from 295 institutions in 43 countries. The journals with the most publications were Blood and Oncotarget. China had the most publications, and Chongqing Medical University and Sun Yat-sen University had the most publications. The author with the highest number of publications was Tschan, Mario P. The main types of research included clinical research, *in vitro* experiments, *in vivo* experiments, public database information, and reviews, and the forms of therapeutic effects mainly focused on genetic regulation, traditional Chinese medicine combination, autophagy inhibitors, and drug targets. The research hotspots of autophagy and AML in the past 17 years have focused on genetic regulation, autophagy inhibition, and targeted drugs. Chemotherapy resistance and mitochondrial autophagy will be the forefront of research.

**Conclusion:** The gradual increase in the literature on autophagy and AML research and the decline after 2022 could be a result of authors focusing more on the type of research and the quality of the literature. The current research hotspots are mainly genetic regulation, autophagy inhibition, and autophagy-related targeted drugs. In future, autophagy will remain the focus of the AML field, with research trends likely to focus more on AML chemotherapy resistance and mitochondrial autophagy.

## 1 Introduction

Acute myeloid leukemia (AML) is a highly lethal malignant blood tumor characterized by abnormal proliferation of myeloid progenitor cells, leading to insufficient production of normal mature hematopoietic cells and bone marrow infiltration, as well as epigenetic changes. The production of normal red blood cells, white blood cells, and platelets is sharply reduced (Hasserjian et al., 2020; Vago and Gojo, 2020), with bleeding, fever, and bone pain as the main clinical manifestations. The prognosis is poor, and some patients also have central nervous system damage, which seriously threatens their lives (Saad et al., 2020). According to statistics, the number of new cases of AML in the US in 2023 is expected to reach 20,380 (34.2% of all new leukaemia cases) and 11,310 deaths (47.7% of all leukaemia deaths) (Siegel et al., 2023). With advances in medical technology, despite the increasing rate of complete remission with AML-inducing chemotherapy, most AML still faces relapsing disease progression, which eventually turns refractory and leads to patient death. Therefore, exploring the pathogenesis of AML plays an important role in the development of drugs for the clinical treatment of AML.

With the mechanism of autophagy discovered by Japanese scientist Yoshinori Ohsumi in 2016 winning the Nobel Prize, research on autophagy has become a hot topic (Tooze and Dikic, 2016). Autophagy is a cellular degradation process that clears dysfunctional, damaged, or aging cells, organelles, and protein transport to lysosomes. Autophagy is regulated through signalling pathways, including the mTOR signalling pathway, the AMPK signalling pathway and the Beclin1-PI3K signalling pathway (He and Klionsky, 2009). These signalling pathways can regulate the initiation and termination of autophagy through the action of catalytic enzymes such as protein kinases or phosphatases. Studies have shown that autophagy mainly involves three types: Macroautophagy, microautophagy, the chaperone-mediated autophagy (Xie and Klionsky, 2007). Among them, Macroautophagy is what we call autophagy, which involves the autophagy-related (ATG) proteins play crucial roles in cancer (Li et al., 2020), and their expression is regulated by Epigenetics (Kumar et al., 2023). Meanwhile, the prognosis and treatment of AML patients are closely related to molecular genetics (Kantarjian et al., 2021). Recent studies have found significant progress in the regulation of cancer diseases, including leukemia, through autophagy (Ichimiya et al., 2020; Du et al., 2021). AML has a complex etiology and diverse clinical grading and classification, and exploring suitable treatment methods through autophagy mechanisms may provide new ideas for potential personalized treatment strategies. In recent years, with the continuous emergence of autophagy research in AML, the publication volume has been increasing year by year.

Bibliometrics, as a cross-science that uses mathematical and statistical methods to analyze knowledge carriers, can reflect the development trend and research hotspots in this field by analyzing the data of countries, authors, periodicals, institutions and keywords in a certain field. In recent years, bibliometrics has been widely used in various fields because of its simplicity, intuition and objective data (Gao et al., 2023; Zhang and Xie, 2023; Yu et al., 2024). However, there is still a lack of related research in the field of autophagy and AML. Therefore, this article will combine bibliometrics to understand the current status and hotspots of autophagy and AML research, draw relevant visualization maps, and predict future development trends to provide a reference basis for subsequent research on autophagy and AML.

## 2 Materials and methods

### 2.1 Data source and literature search

The literature data was obtained from the Web of Science Core Collection (WoSCC) database, and the search time was from 1 March 2003 to 1 March 2023. The search formula used was TS =((acute myeloid leukemia) OR AML) AND TS=(autophagy).

### 2.2 Data screening

#### 2.2.1 Inclusion criteria

(1) Literature related to autophagy and acute myeloid leukemia; (2) Literature published in English; (3) Literature types include clinical trial studies, *in vitro* experimental studies, *in vivo* experimental studies, public database analysis studies, reviews, etc.; (4) Literature with complete bibliographic information (including title, country, author, keywords, source).

#### 2.2.2 Exclusion criteria

(1) Conference papers, newspapers, patents, achievements, health and popular science literature, etc.; (2) Duplicate publications.

#### 2.2.3 Data standardization

After screening, the literature was exported in Refworks and plain text formats. Special symbols were removed. Keyword names were standardized, for example, “acute myelogenous leukemia” was merged into “acute myeloid leukemia”, and “breast cancer” was determined as “cancer”. Then, the Data Import/Export function in CiteSpace software was used to convert the format of the retrieved literature.

#### 2.2.4 Data analysis

Excel, VOSviewer 1.6.18, CiteSpace (6.1.R3), RStudio (R package bibliometrix), and Scimago Graphica software were used to analyze the included literature for publication volume, journals, core author cooperation, and keyword analysis. The time slice period was set to January 2007 to March 2023 (from the first publication of literature within 20 years until now), and the year per slice was set to 1 year. Co-occurrence analysis of keywords and institutions was selected as node types. The pruning method was set to the default value. Microsoft Office Excel could be used for annual publication volume, countries production over time and literature research classification statistics. Moreover, RStudio (R package bibliometrix) was being used to draw some visual map to analyze Journal Sources, countries, international collaboration, co-cited references the frequency keywords and authors. CiteSpace.6.1.R3 was used to analyze institutions collaboration, the centrality of institutions, and the clusters and burst terms of keywords. VOSviewer 1.6.18 was used to analyze co-cited references. Furthermore, Scimago Graphica was used to analyze authors and the frequency keywords.Co-occurrence analysis, clustering analysis, and burst analysis were conducted on the included data, and the knowledge map was generated and adjusted for visualization analysis.

## 3 Results

### 3.1 Literature search and selection results

**FIGURE 1:**
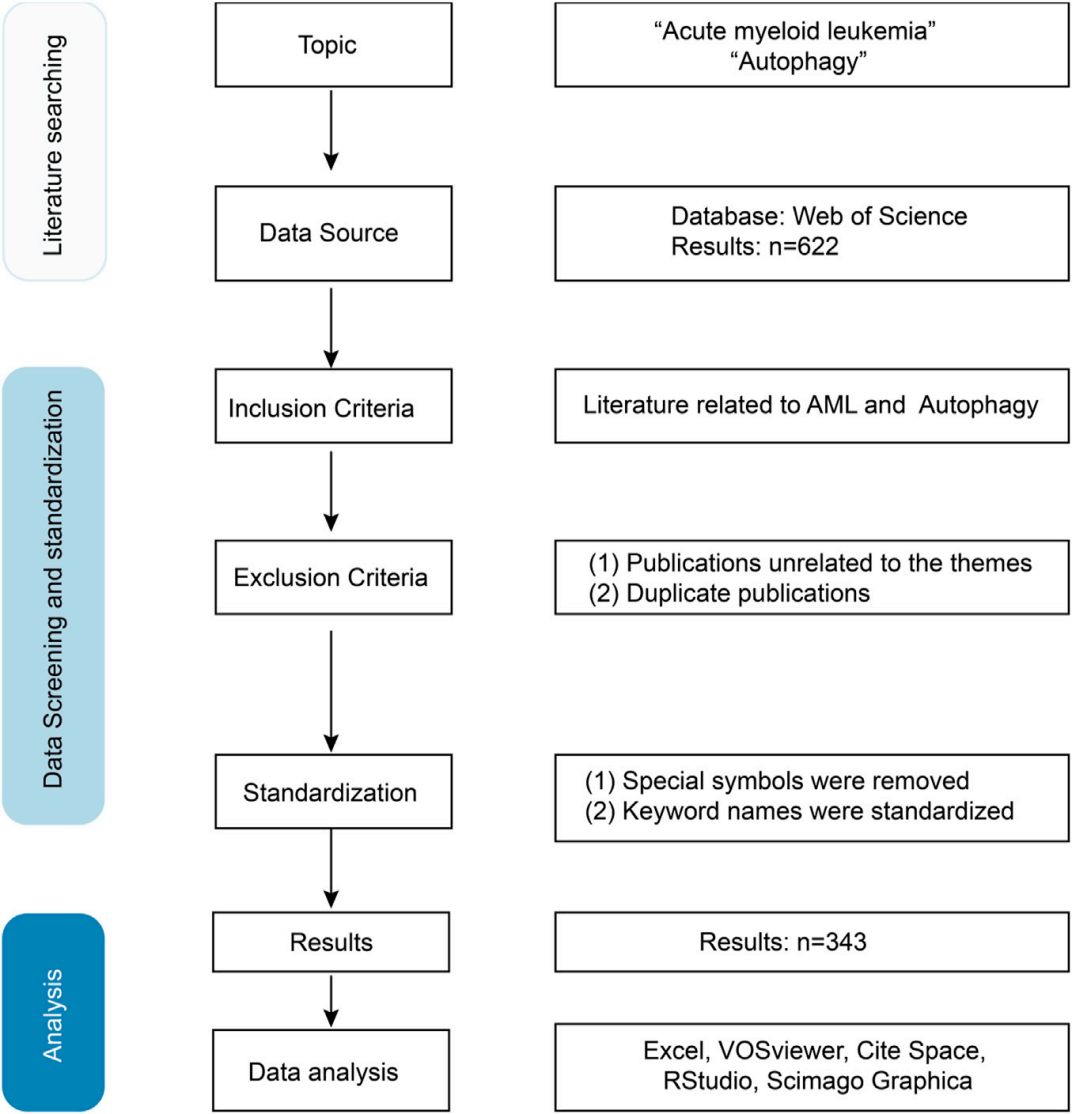
The flowchart of literature data screening.

A total of 622 articles were retrieved through the literature search. After removing duplicates and screening for eligibility, 343 articles were selected based on inclusion criteria. The specific screening process is shown in Figure 1.

### 3.2 Analysis of publication year and journal sources

**FIGURE 2:**
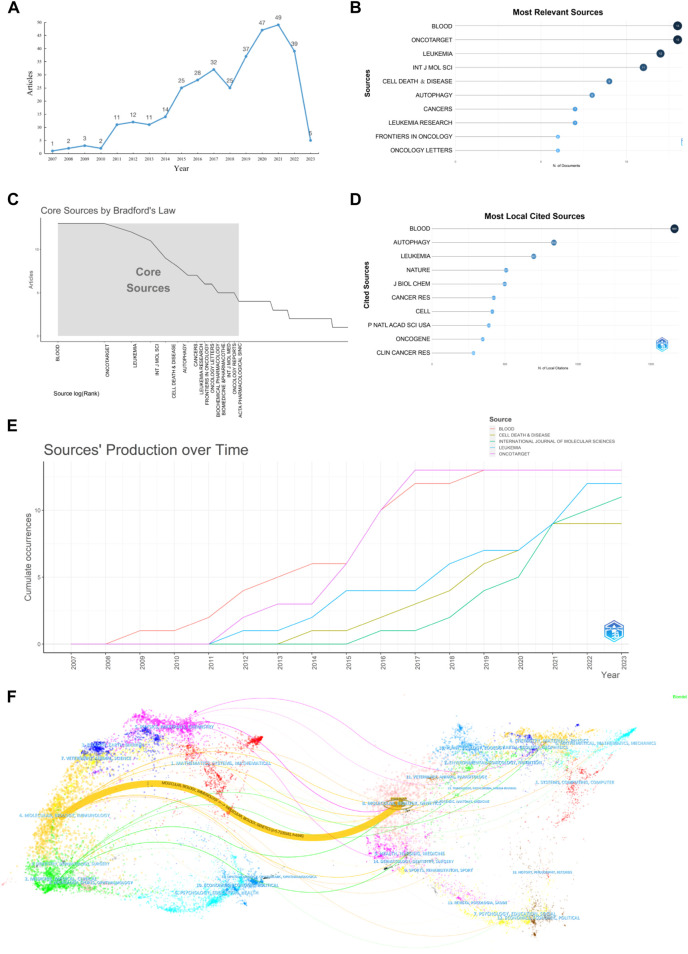
Visualization of publication quantity and journal sources. **(A)** Annual distribution of publication quantity; **(B)** Top 10 journals in terms of publication quantity; **(C)** Core journals determined according to Bradford’s law; **(D)** Top 10 journals cited in the publications; **(E)** Annual production trends of the top 5 journals in terms of publication quantity; **(F)** Dual map of journal citation relationships, with clustering of citing and cited journals.

Analysis of the publication trends in the field of autophagy and AML revealed a relatively small but increasing number of publications, as shown in Figure 2A. The first publication appeared in 2007, and the number of publications reached a peak in 2020 (53 articles). From 2007 to 2010, the number of publications was consistently below 10 per year, representing the initial stage of research in this field. From 2011 to 2017, there was a steady increase in the number of publications, and from 2018 to 2021, there was a rapid increase. Although there was a slight decrease in 2018 and 2022, the overall trend in publication volume is still increasing. Based on the analysis of literature research types, it was found that in 2018 and 2022, the number of *in vivo* experimental studies decreased, while clinical studies slightly increased, and the quality of publications gradually improved. It is speculated that the change in research subjects and emphasis on paper quality may have led to a decrease in the number of publications. A total of 169 journals published articles related to autophagy and AML, with Blood and Oncotarget being the top two journals in terms of publication volume (13 articles), as shown in Figure 2B. According to Bradford’s law, the top 15 core journals in this field are Blood, Oncotarget, Leukemia, International Journal of Molecular Sciences, Cell Death & Disease, Autophagy, Cancers, Leukemia Research, Frontiers in Oncology, Oncology Letters, Biochemical Pharmacology, Biochemical & Pharmacothe, International Journal of Oncology, Oncology Reports, and Acta Pharmacologica Sinica, as shown in Figure 2C. Blood (1,661 articles) is the most frequently cited journal in the field of autophagy and AML, as shown in Figure 2D. Figure 2E shows that Blood and Oncotarget had the highest number of publications in 2016 (4 articles), while International Journal of Molecular Sciences had the highest number of publications in 2021 (4 articles). The overlapped journal network analysis demonstrated the citation relationship between journals and their cited journals. The left part in Figure 2F showed the cluster of citing journals, while the right part showed the cluster of cited journals. The orange path indicated that the research from Molecular Biology Genetics journals was most likely to be cited by the Molecular Biology Immunology journals.

### 3.3 Visual analysis of international collaboration

**FIGURE 3:**
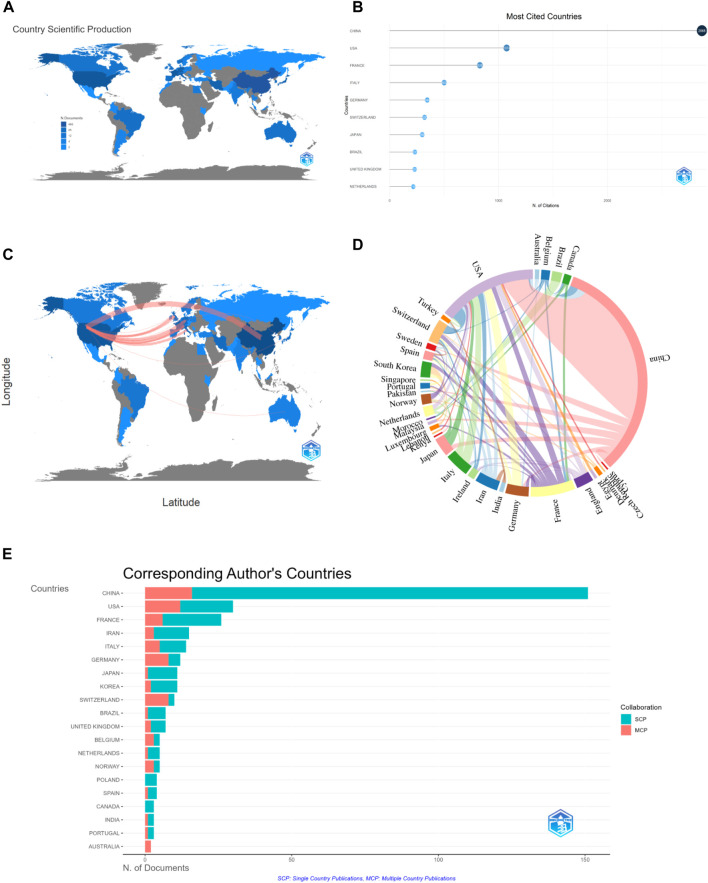
Visual mapping of the origin of the countries of publication. **(A)** Distribution of national article issuance; **(B)** top 10 cited countries in terms of article issuance; **(C)** distribution of inter-country collaboration; **(D)** chordal map of inter-country collaboration distribution; **(E)** top 20 countries in terms of number of corresponding authors.

43 countries have published research literature related to autophagy and acute myeloid leukemia, as shown in Figure 3A. The top ten countries in terms of publication volume are China (158 papers), United States (69 papers), France (31 papers), Italy (17 papers), Germany (16 papers), Switzerland (16 papers), Iran (16 papers), Japan (13 papers), England (12 papers), and South Korea (11 papers), as shown in Figure 3B. China has been cited the most (2,868 papers), followed by the United States and France. Figures 3C, D show the collaboration between different countries in the field of autophagy and AML research. The thicker the line between two countries, the more collaboration there is between them. Therefore, we can infer that the collaboration between the United States and other countries is closer. In Figure 3E, SCP represents articles whose authors are all from the same country, while MCP represents articles whose authors are from multiple countries, indicating international collaboration. Therefore, it can be inferred that China has the most collaborative authors with other countries in this field, but internal collaboration within China is still greater.

### 3.4 Visualization analysis of authors and institutions collaboration

**FIGURE 4:**
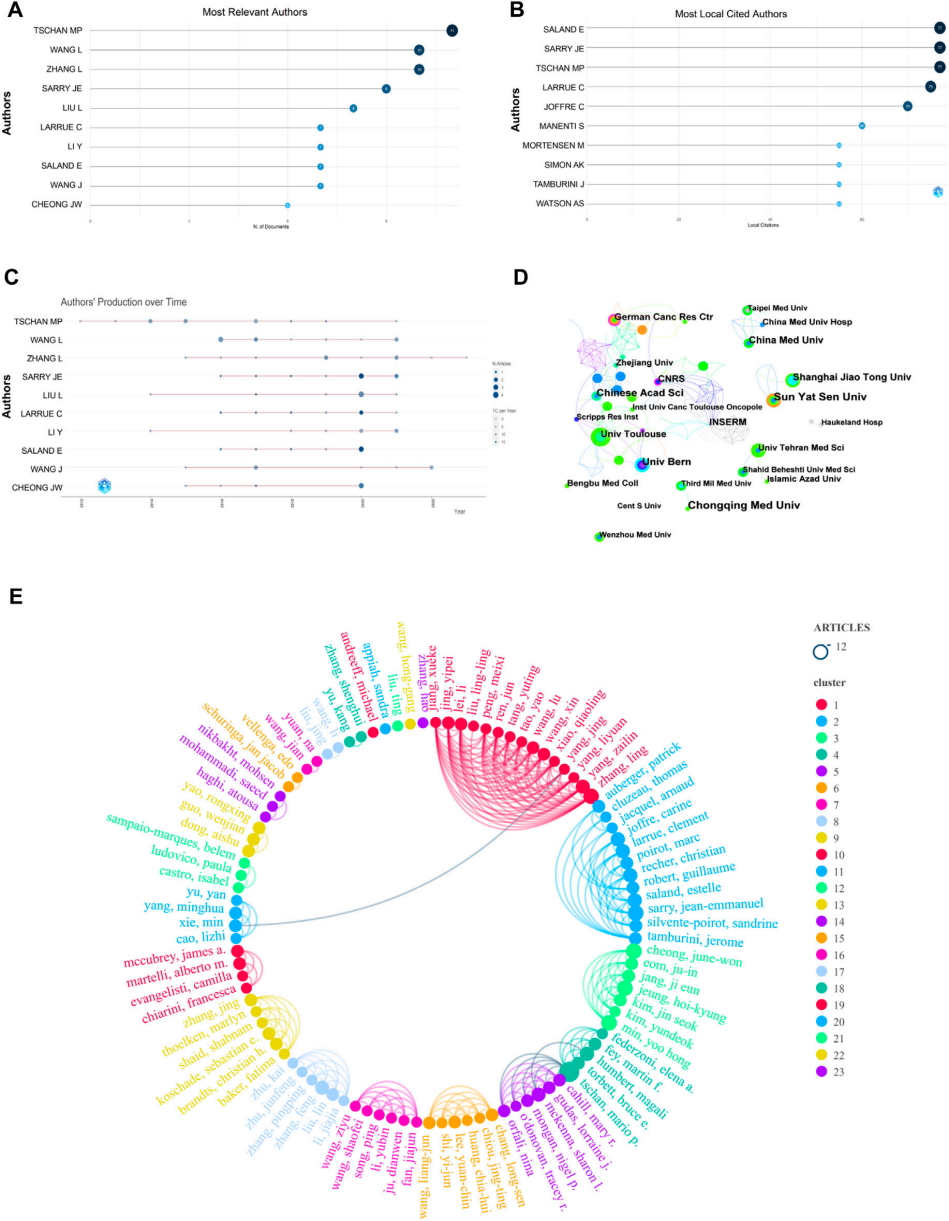
Visual mapping of authors and institutions. **(A)** Top 10 authors in terms of publications; **(B)** top 10 cited authors in terms of publications; **(C)** annual publication trends of top 10 authors in terms of publications; **(D)** distribution map of inter-institutional collaborations; **(E)** network map of core-author collaborations.

The visualization analysis of 343 Chinese literature articles included a total of 2,323 authors. The author with the most published articles is Tschan, Mario P from the University of Bern (11 articles). The top 10 authors and their annual publication changes are shown in Figures 4A, C. The authors with the most cited articles are Saland, Estelle and Sarry, Jean-Emmanuel from Université de Toulouse, and Tschan, Mario P from the University of Bern (77 articles), as shown in Figure 4B. According to the Price’s Law formula ( $\text{Mp}=0.749*\sqrt{N_{\text{pmax}}}$ , the number of core authors > Mp articles), where Mp = 2.37, the authors with three or more published articles are considered as core authors. This study has 99 core authors. In Figure 4E, each node represents an core author, and the size of the node indicates the number of articles published by the author. The more connections a node has, the closer the collaboration relationship between the authors. Different colors represent the relationship between different collaborative groups. The results show that there are four academic groups with relatively close cooperation in this field, including Tschan Mario P, Sarry Jean-Emmanuel, Cheong June-won, and Zhang Ling. Their research contents are experimental studies on the anti-leukemia effects related to autophagy genes (Jin et al., 2018), dendritic protein A (Serhan et al., 2020), autophagy inhibitors (Kim et al., 2015) and NPM1 mutations (Tang et al., 2021).

The collaboration network of research institutions includes 295 nodes and 455 connections, representing a total of 295 institutions. The top 10 institutions in terms of the number of articles published are listed in Table 1. The research institution with the most published articles is Chongqing Medical University and Sun Yat-sen University (9 articles), and the institution with the highest centrality is CNRS. Betweenness Centrality represents the importance of the node in the network. Each node in the figure represents an institution, and the larger the text, the more articles the institution has published. The connections represent the collaboration relationships between institutions. As shown in Figure 4D, the institutions that have closer collaboration include Chinese Academy of Sciences, German Cancer Research Center, Université de Toulous, University of Bern, and CNRS. The results indicate that the research institutions are mostly universities from various countries, and collaborations between institutions in Europe and Asia are more frequent.

**TABLE 1:** Top 10 institutions in terms of number of publications (left), top 10 institutions in terms of centrality (right).

No.	Institutions	Publications	No.	Institutions	Centrality
1	Chongqing Medical University	9	1	CNRS	0.11
2	Sun Yat-sen University	9	2	German Cancer Research Center	0.10
3	Chinese Academy of Sciences	8	3	Albert Ludwigs Univ Freiburg	0.09
4	University of Bern	8	4	INSERM	0.06
5	Shanghai Jiao Tong University	7	5	Heidelberg Univ	0.04
6	China Medical University	7	6	Harvard Med Sch	0.04
7	Université de Toulouse	6	7	Chinese Academy of Sciences	0.04
8	German Cancer Research Center	6	8	Inst Univ Canc Toulouse Oncopole	0.02
9	INSERM	6	9	University of Bern	0.01
10	CNRS	6	10	Université de Toulouse	0.01

### 3.5 Analysis of cited literature

**FIGURE 5:**
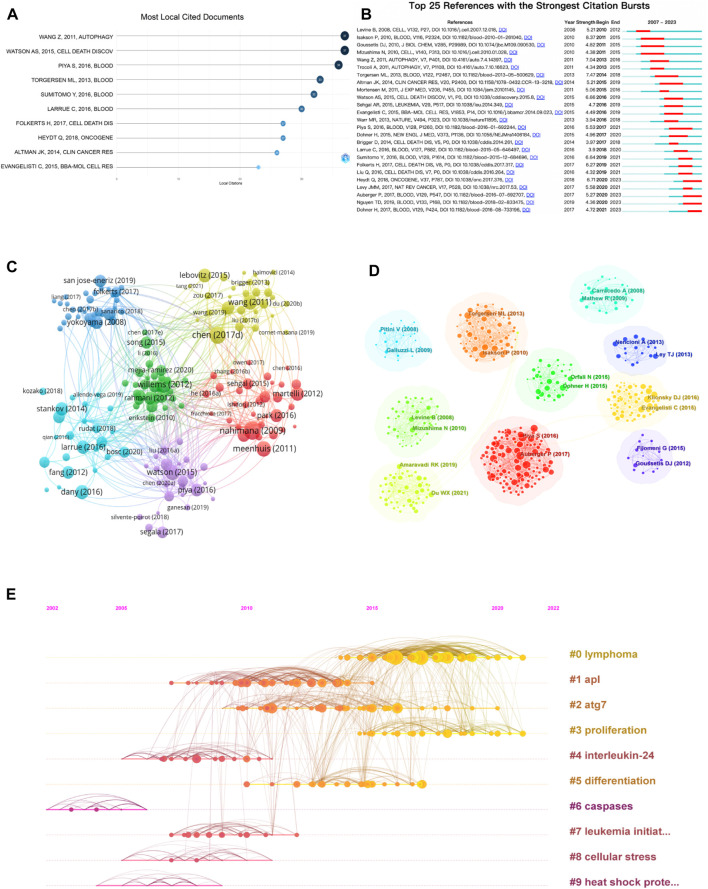
Visual mapping of cited literature. **(A)** Top 10 cited literature; **(B)** emergent analysis of cited literature; **(C)** thematic coupling analysis of research literature; **(D)** clustering analysis of cited literature; **(E)** timeline analysis of cited literature.

According to Figure 5A, the three most cited papers are: WANG Z, 2011, AUTOPHAGY (Wang et al., 2011); WATSON AS, 2015, CELL DEATH DISCOV(Watson et al., 2015; Piya et al., 2016, BLOOD (Piya et al., 2016). Among the top 10 cited papers, four are about research on targeting autophagy inhibition. Citation emergence refers to a sudden increase in citation frequency in a short period of time, which can show the research hotspots in a certain period of time and the transfer of research hotspots in different periods of time. It is also used to judge the development dynamics and trends of research hotspots. Figure 5B shows the top 25 cited references with citation emergence, and four papers have had a significant increase in citation frequency in recent years. This may indicate that the future trend of autophagy research in the AML field will focus on the mechanisms of autophagy, targeting autophagy for anti-leukemia treatment (Heydt et al., 2018), the role of autophagy in leukemia resistance (Auberger and Puissant, 2017), and mitochondrial autophagy (The Duy et al., 2019). The research literature topic coupling analysis is shown in Figure 5C, which obtained six clusters: the purple cluster included most of the articles on protective autophagy; the blue cluster focused on autophagy inhibition; the red cluster mainly concentrated on biological targets; the yellow cluster included most of the articles on targeting autophagy inhibition; the pink cluster mainly focused on genetic informatics; the green cluster mainly concentrated on drug resistance. The citation clustering is shown in Figures 5D, E. Clusters 0 (red), 2 (yellow), and 3 (yellow-green) showed that lymphoma, ATG7 (an autophagy-related gene), and proliferation are hot research topics that continue to this day; clusters 4 interleukin-24, 6 cysteine protease, and 9 heat shock protein are related to treatment targets; clusters 0 lymphoma, 2 acute myeloid leukemia, and 7 leukemia stem cells reflect that leukemia research has multiple directions; clusters 3 proliferation, 5 differentiation, and 8 cellular stress are related to the corresponding mechanisms.

### 3.6 Keyword visualisation analysis

#### 3.6.1 Keyword co-occurrence analysis

**FIGURE 6:**
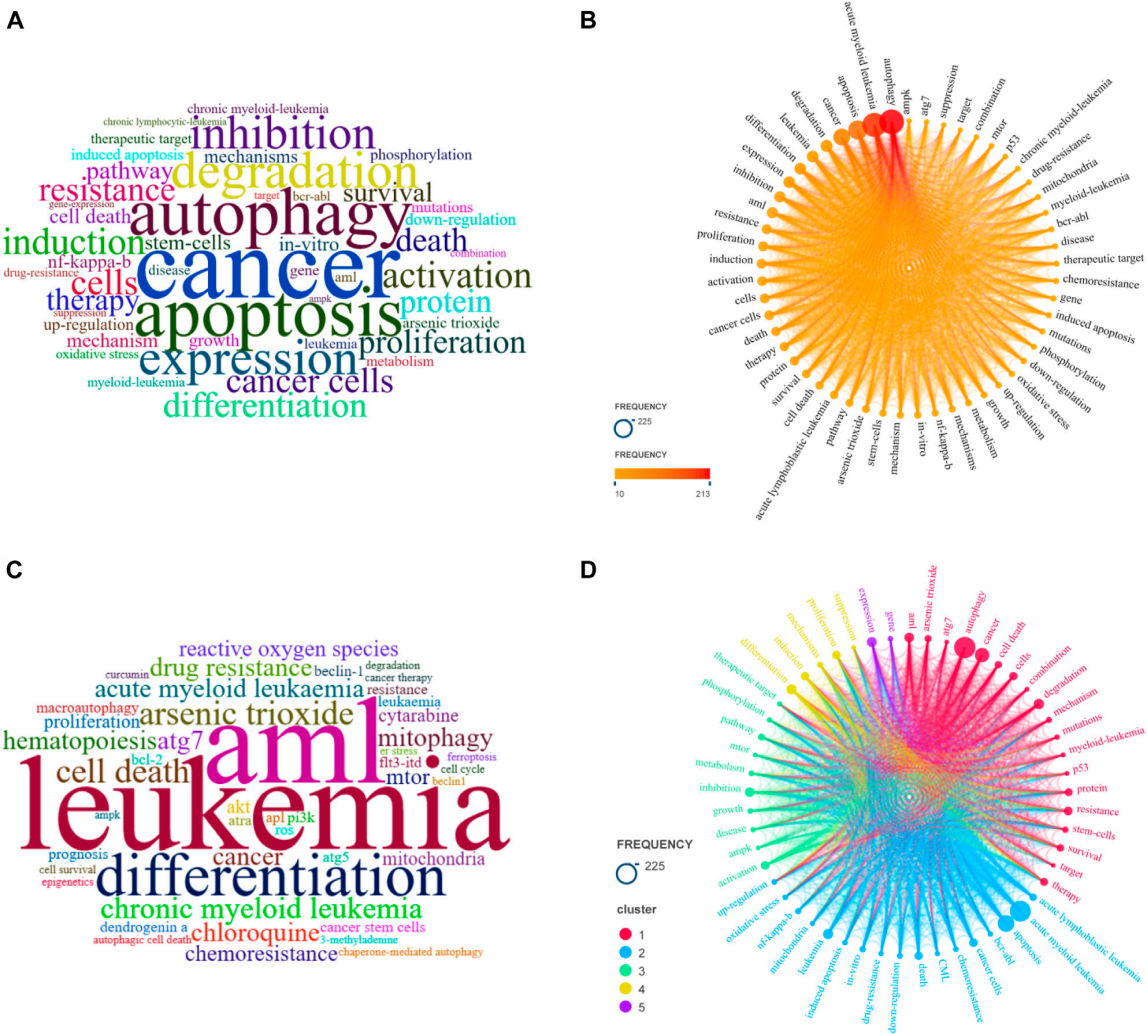
The bibliometric analysis of the keywords. **(A)** Word clouds of keywords plus in local clusters; **(B)** Keyword frequency distribution; **(C)** Word clouds of author keywords; **(D)** Keyword co-occurrence analysis.

In this study, there were 55 keywords that co-occurred more than 10 times, as shown in Figures 6A–C. The co-occurrence analysis in Figure 6D revealed that the study focused on *in vitro* experiments, *in vivo* experiments, mechanism of action, drugs, and gene regulation. *In vitro* experiments mainly involved cell experiments, with common cell models including HL-60 cells, NB4 cells, and KG-1a cells. *In vivo* experiments and clinical trials were more common in mouse models. In terms of mechanism of action, the study mainly focused on regulating gene targets and signaling pathways to induce apoptosis, inhibit autophagy, and exert anti-leukemia effects in cell and mouse models. Commonly used drugs and drug targets included colony stimulating factor, arsenic trioxide, histone deacetylase inhibitor, beclin 1 (a key regulatory protein of autophagy), and BCR-ABL inhibitors.

#### 3.6.2 Keyword burst analysis

By analyzing the emerging keywords, the research hotspots in the field and their changes over time can be intuitively displayed, and research trends can be predicted. As shown in Figure 5A, some scholars began to explore the relationship between autophagy and AML-related signaling pathways in 2007, mainly through the mTOR and MAPK signaling pathways. Between 2012 and 2021, research on autophagy and AML focused on *in vitro* experiments and the effects of related inhibitors, with BCR-ABL kinase inhibitors, dendritic cell factor A (DDA), oncogene proteins, and arsenic trioxide as representatives. From 2021 to 2023, gene mutations and mechanism of action remained research hotspots, with ATG5, ATG7, and FLT3-ITD as related genes. Based on this, it is predicted that chemotherapy resistance, specific mechanisms of action, and gene mutations will be the future research hotspots in the field of autophagy and AML after 2023.

**FIGURE 7:**
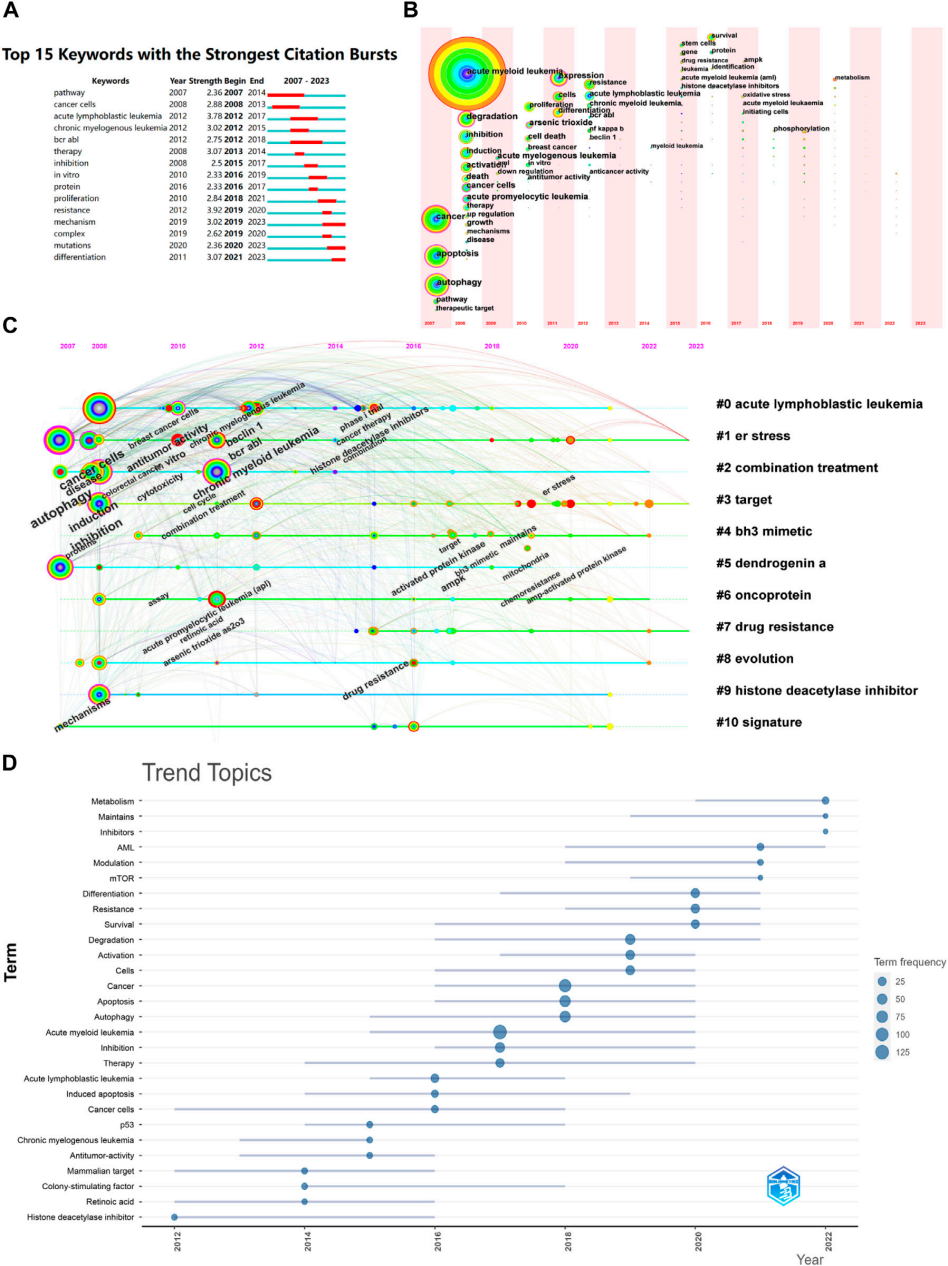
Keyword bursts and keyword time zone analysis. **(A)** Keyword bursts; **(B)** Keyword timeline; **(C)** Keyword time zones; **(D)** Trend topics of research of AML and autophagy from 2012 to 2022.

The visualization analysis of time zone for keywords can analyze the research hotspots from a temporal perspective based on keyword co-occurrence, and display the development and changes of keywords in chronological order, further exploring the research hotspots, evolution history, and frontiers of this field. As shown in Figures 7B–D, the results indicate that the research hotspots in this field have evolved in three time stages: (1) from 2007 to 2009, only basic keywords such as AML, tumor, autophagy, signaling pathway, cell apoptosis, and combination therapy appeared, indicating that the academic community has started to study this field but the enthusiasm is lacking; (2) from 2010 to 2015, keywords such as *in vitro* experiments, arsenic trioxide, dendritic cell cytokine A, etc. began to emerge, and cell research began to become a trend; (3) from 2015 to 2023, gene regulation, drug resistance, and targeted therapy have successively become hotspots, indicating that the research has shifted towards molecular targeted mechanism research.

## 4 Discussion

This article uses software such as CiteSpace, VOSviewer, and R language to conduct a visual analysis of 343 papers on autophagy in the field of acute myeloid leukemia (AML) in the WOS database from 2003 to 2023. By drawing a knowledge map, it intuitively displays the current status, hotspots, and development trends of the literature publication situation, journal sources, research authors, institutional cooperation networks, and research fields in this field in the past 17 years.

### 4.1 Research status

In terms of the annual number of publications, the overall number of publications in this field is on the rise. The main countries of publication are China, the United States, and France. The journals with the highest number of publications are Blood and Oncotarget. The author with the highest number of publications is Tschan, Mario P, and the author cooperation network is mainly formed by cooperation teams represented by Tschan Mario P, Sarry Jean-Emmanuel, Cheong June-won, and Zhang Ling. Among them, the research institutions that published the most papers are Chongqing Medical University and Sun Yat-sen University. The research institutions are mostly universities from various countries, and institutional cooperation is more frequent between Europe and Asia. These results indicate that compared with research results in other fields, there are relatively few research achievements on autophagy in AML. In the future, it is necessary to further increase international and intercontinental cooperation between institutions, share research results, and facilitate the in-depth development of this field.

**FIGURE 8:**
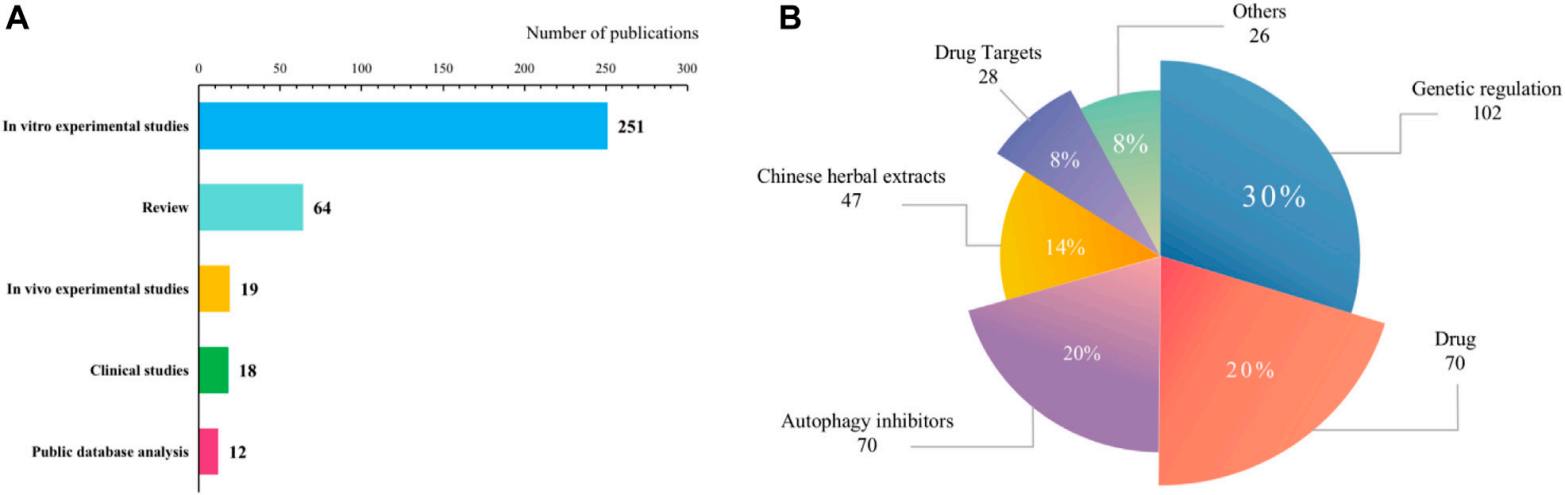
Classification of studies in the literature. **(A)** Type of literature; **(B)** Direction of treatment.

The results of literature research classification and keyword clustering analysis show that the research types of autophagy and AML mainly include clinical research, *in vitro* experiments, *in vivo* experiments, public information database information, and reviews. As shown in Figure 8, the forms of autophagy involved in AML treatment are mainly genetic regulation, drug combination, autophagy inhibitors, drug targets, etc. The research hotspots of gene regulation mainly focus on autophagy-related genes such as Bcl-2 (Tang et al., 2021), p35 (Huang et al., 2023), Beclin 1 (Radwan et al., 2016), and ULK1 (Tang et al., 2021) Autophagy inhibitors mainly include chloroquine (CQ) (Gronningsaeter et al., 2021), hydroxychloroquine (HCQ) (Kim et al., 2015) 3-methyladenine (3 MA) (Song et al., 2022), and histone deacetylase (HDAC) inhibitors (San Jose-Eneriz et al., 2019). The drug targets involve casein kinase 1*α* (CK1*α*) (Xu et al., 2020), lysine-specific demethylase 1 (LSD1) (Liu et al., 2017), asparagine synthetase (Song et al., 2015), etc. In addition, related Chinese herbal medicines and extracts are also gradually emerging, such as salvianolic acid IIA (Song et al., 2022), arsenic trioxide (Liu et al., 2020), curcumin (Tseng et al., 2019), etc. Based on visual analysis, high-frequency keywords such as autophagy inhibitors, genetic regulation, and chemotherapy resistance indicate that experimental research on molecular targeted drugs is still one of the research hotspots in this field.

### 4.2 Research hotspots

Keywords are crucial vocabulary that express the core themes of a literature. Through keyword analysis, this study found that frequently occurring keywords include signaling pathways, gene expression, apoptosis, and autophagy inhibition. Further coupled with co-occurrence and citation clustering analysis, as shown in Figures 5C–E, the blue and yellow clusters of research topics are mainly focused on autophagy inhibition, while the red cluster and research contents related to interleukin-24, caspase-6, and heat shock proteins are mainly focused on related therapeutic targets. The clustering of ATG7 (#2) indicates that autophagy-related genes are a hot research topic in this field. This suggests that over the past 17 years, research on autophagy and AML has focused on genetic regulation, autophagy inhibition, and targeted drugs.

### 4.3 Genetic regulation

In recent years, with the development of molecular informatics, research on genetic regulation in AML has become increased. Sumitomo Y et al. found through *in vitro* experiments that genetic suppression of autophagy-related genes (Atg5, Atg7) induced by tamoxifen led to autophagy inactivation, thereby prolonging the survival of leukemia mouse models and eliminating leukemia stem cells, indicating that knockout of autophagy-related genes can serve as a new approach for AML treatment (Sumitomo et al., 2016). In addition, Bollaert E et al. found that microRNAs play an important role in AML. Experimental verification showed that miR-5a-15p can eliminate the chemoresistance of AML cells induced by daunorubicin-induced autophagy, and miR-5a-15p may be a promising therapeutic target for chemotherapy-resistant AML patients. (Bollaert et al., 2021). Zhang H et al. found that targeted autophagy dependent on ATG7 and ATG2B is a key mechanism by which miR-143 makes AML cells sensitive to cytarabine, suggesting that it is a potential therapeutic target for AML treatment (Zhang et al., 2020). Jiang XK et al. found through bioinformatics databases and experimental verification that overexpression of miR-148b-3p can inhibit the *in vitro* proliferation and autophagy of AML cells by targeting ATG14, thereby exerting an anti-leukemia effect. (Jiang X. et al., 2021). Based on this, autophagy-related genes and miRNAs may be important targets for the therapeutic effects of autophagy in AML, but their specific mechanisms require further study.

### 4.4 Autophagy inhibition

Autophagy inhibition has been repeatedly mentioned in the keywords of autophagy and AML research. Most scholars believe that autophagy has a bidirectional regulatory effect in leukemia treatment (Seo et al., 2022). In the past decade, some articles on the treatment of AML with autophagy inducers have been published. For example, Dihydroartemisinin (DHA) plays an anti-leukemic effect by triggering ferroptosis in leukemic cells, and DHA induced autophagy by regulating the activity of AMPK/mTOR/p70S6k signaling pathway, which plays a key role in ferritin degradation, ROS accumulation and ferroptotic cell death (Du et al., 2019). Vilimanovich U and others have shown that statins and agents that selectively reduce intracellular cholesterol levels induced autophagy in leukemic cells.The observed autophagic response was associated with the inactivated the main autophagy suppressor mTOR and its substrate ribosomal p70S6 kinase (p70S6K). This might be potentially useful in anti-leukemic therapy (Vilimanovich et al., 2015). But current research tends to achieve anti-leukemia effects by inhibiting autophagy. Gronningsaeter IS and others have shown that the autophagy inhibitory effect of the antimalarial drug chloroquine can be used for acute myeloid leukemia. Through *in vitro* experiments on 81 AML patients, chloroquine was found to reduce the viability and proliferation of AML cells in most patients, exhibiting anti-leukemia activity (Gronningsaeter et al., 2021). Jang JE and others found that the AMPK-ULK1 pathway plays a crucial role in autophagy of stem cells, and targeting the AMPK-ULK1 pathway or inhibiting autophagy can overcome drug resistance and enhance the apoptotic response of cells, which may be an effective treatment strategy against BET inhibitor resistance in AML (Jang et al., 2017). Wu JQ and others found that matrine can exert an anti-leukemia effect by inducing apoptosis and autophagy inhibition through the inhibition of signaling pathways such as Akt and mTOR (Wu et al., 2017). These studies indicate that autophagy inhibition may be a potential strategy for AML treatment, but further research is needed to provide strong support for it.

### 4.5 Targeted drugs

With more and more gene targets and autophagy targets being proposed, corresponding therapeutic drugs are emerging one after another. Benjamin DN et al. found that all-trans retinoic acid combined with valproic acid promotes differentiation of leukemia cells by inhibiting autophagy regulatory factors ATG7 and TFEB (Benjamin et al., 2022). Xiao J et al. found through cell experiments that quercetin regulates the AMPK/mTOR signaling pathway to inhibit cell differentiation, induce cell apoptosis and autophagy, and exert its anti-leukemia effects (Xiao et al., 2022). Chen LY et al. found that low-dose cytarabine can induce autophagy in AML cells through downregulating the Akt-mTOR signaling pathway and exert an anti-tumor effect (Chen et al., 2017). Liu XJ et al. found that arsenic trioxide has a good therapeutic effect on an AML mouse model. The results show that arsenic trioxide exhibits significant anti-cancer activity in FLT3-ITD AML cells and induces autophagy degradation of FLT3-ITD mutated protein to exert anti-leukemia effects (Liu et al., 2020). Jiang L et al. found that knocking down Beclin-1 expression to inhibit autophagy increases borrelidin-induced apoptosis in acute promyelocytic leukemia (APL) cells, indicating that the combination of borrelidin and autophagy inhibition may be a potential therapeutic strategy for APL (Jiang et al., 2021a). Haghi A et al. found that the combination of sorafenib and arsenic trioxide significantly increases the number of apoptotic cells in AML cell lines, which may be related to autophagy (Haghi et al., 2020). In summary, combination therapy seems to have greater therapeutic potential in targeted therapy for AML.

### 4.6 Research trends

Based on the visualization analysis of keywords and cited references using multiple methods, it was found that the study of chemotherapy resistance and mitochondrial autophagy in AML is a current research hotspot and also a future development trend.

### 4.7 Chemotherapy resistance

Chemotherapy resistance is a major challenge in clinical treatment of AML. Currently, clinical treatment of AML mainly relies on chemotherapy, radiation therapy, and stem cell transplantation, which have improved the cure rate and relief rate to some extent (Liu, 2021). However, disease recurrence caused by chemotherapy resistance has become the primary reason for treatment failure. In recent studies, it has been found that many clinical drugs can activate cell protective autophagy, including Beclin 1/Bcl-2 inhibitors (Chiang et al., 2018) and BET inhibitors (Jang et al., 2017), and this cytoprotective effect can increase chemotherapy resistance (Chen et al., 2019; Saulle et al., 2023). In the above cases, targeting autophagy combined with chemotherapy as an emerging research direction may provide new treatment strategies. A study by Auberger P et al. found that ULK1-induced autophagy activation is one of the potential mechanisms of anthracycline resistance in AML, and selective targeting of ULK1 may be a promising treatment strategy to improve the sensitivity of AML to cytarabine/anthracycline combination chemotherapy (Auberger and Puissant, 2017). Another article published in 2022 by Xu D et al. also suggested that targeting autophagy could be an effective approach to overcome AML drug resistance. The study found that autophagy activation weakened the anti-leukemia effect of FLT3 inhibitors, while chloroquine inhibition of autophagy significantly enhanced the anti-leukemia effect of FLT3 inhibitors (Xu et al., 2022). In addition, circPAN3 was found to promote AML drug resistance by regulating autophagy and affecting the expression of apoptosis-related proteins, indicating that circPAN3 may be a potential target for AML drug resistance treatment (Shang et al., 2019). Meanwhile, Ge CY et al. found that salidroside enhances the anti-tumor effect of imatinib in AML by activating autophagy and increasing its cytotoxicity against acute monocytic leukemia. (Ge et al., 2019). These studies indicate that targeting autophagy and combination therapy have achieved some results *in vitro* experiments on AML drug resistance, but there is still a lack of safety evaluation and clinical validation.

### 4.8 Mitophagy

Mitophagy is a form of selective autophagy that maintains mitochondrial integrity and cellular homeostasis by clearing dysfunctional mitochondria from the cytoplasm (Lu et al., 2023). Dysregulation of cellular homeostasis is closely associated with cancer diseases, including AML. Leukemia stem cells (LSCs) have always been considered as one of the factors that promote the genesis of AML as well as relapse following chemotherapy. In addition, selective autophagy is very important for the life-long maintenance of hematopoietic stem cells (HSCs), which demonstrated that mitophagy plays an indispensable role in the survival of leukemia-initiation cells (LICs) during AML development and progression (Li et al., 2021). Researchers have demonstrates that intrinsic over-expression of the mitochondrial dynamics regulator FIS1 mediates mitophagy activity that is essential for primitive AML cells (Pei et al., 2018). Therefore, targeting mitophagy in AML may become a promising target for anti-leukemic therapy. In recent years, the biological role of mitophagy in the development of AML has become a hot topic of interest among scholars and pharmaceutical research and development companies. Dany M et al. found that targeting FLT3-ITD can induce AML cell death by mediating ceramide-dependent mitophagy, providing a new strategy for AML treatment. (Dany et al., 2016). Hao BB et al. found that CC-885 (a derivative of thalidomide) can enhance the sensitivity of AML cells to the mitochondrial-targeting drug venetoclax, suggesting that combined therapy with CC-885 and mitochondrial-targeting drugs may be a treatment strategy for AML patients (Hao et al., 2020). Fay H et al. found that residual leukemia cells after chemotherapy in a hypoxic environment can increase mitochondrial autophagy in certain cell types by upregulating BNIP3 to protect surviving AML cells. However, subsequent research results suggest that treatment with autophagy inhibitors may block mitophagy and induce AML cell death under hypoxia, providing a new approach to targeting minimal residual disease in AML treatment (Fay et al., 2019). Rodrigo R et al. found that drugs targeting mitochondria can selectively eliminate AML cells, and BNIP3L or SQSTM1 (mitophagy-related genes) may be useful biomarkers for identifying AML patients suitable for mitochondrial-targeted therapy (Rodrigo et al., 2019). In summary, the study of mitophagy in AML in the academic community is still in its early stages, and future research can focus on targeting autophagy and mitophagy at the molecular level to further elucidate the molecular mechanisms of mitophagy and develop targeted small molecule drugs, actively exploring new ideas and methods for AML treatment.

### 4.9 Limitation

The limitations of the current research on the role of autophagy in AML treatment are mainly focused on *in vitro* experiments, including gene regulation, autophagy inhibition, and targeted drugs. However, there is a lack of systematic research, and in the future, it is necessary to combine techniques such as bioinformatics and molecular targeting regulation to provide strong theoretical support for the treatment of AML. Compared with traditional reviews, this article can predict future research trends and directions through visualization. However, this research method still has certain shortcomings. This article only searched for relevant literature in the WOS database, which may have missed other relevant literature in other databases, and cannot fully reflect the research overview of this field. In the future, the scope of literature search should be expanded, and high-quality basic research and multicenter real-world data research should be included as much as possible to promote the development of the role of autophagy in AML and provide new ideas for future research on autophagy and AML.

## 5 Conclusion

This study used bibliometric tools such as CiteSpace, VOSviewer, and R language to perform a visual analysis of literature related to autophagy and acute myeloid leukemia (AML).The study visually show the current status, hotspots, and development trends in this field over the past 17 years, and offer discusses and analyzes the related research. Through the annual publications analysis, it is found that autophagy-related research in AML treatment is still in the development stage. The journal BLOOD has the most publications and cited references, indicating its significant influence in this field. The visual analysis shows that the hotspots of autophagy and AML research are focused on genetic regulation, autophagy inhibition, and targeted drugs, with future research trends being the study of chemotherapy resistance and the role of mitochondrial autophagy mechanisms in AML. Through literature analysis, it is found that the study of chemical resistance and mitochondrial autophagy may provide new ideas for the potential study of autophagy involved in AML therapy.This study is timely to promote future research directions and trends in this field, but there are still limitations. Future studies should expand the database search scope and collect more high-quality and representative articles to provide strong data support for research in autophagy and AML.

## Data Availability

The raw data supporting the conclusion of this article will be made available by the authors, without undue reservation.
